# Comprehensive Review on Current Controversies and Debate in Prostate Artery Embolization

**DOI:** 10.5152/tud.2022.21337

**Published:** 2022-05-01

**Authors:** Drew Maclean, Charles Timothy Francis Bryant, Ganesh Vigneswaran, Timothy JC Bryant, Mark Harris, Bhaskar Somani, Sachin Modi

**Affiliations:** 1 Department of Interventional Radiology, University Hospital Southampton, Southampton, UK; 2 Department of Urology, University Hospital Southampton, Southampton, UK

**Keywords:** Prostate, artery, embolization, BPH, controversy

## Abstract

Prostate artery embolization is emerging as one of the most effective therapies amidst a new era of minimally invasive benign prostate hyperplasia treatment and technology. However, several current controversies remain unanswered which could impact the widespread adoption of this novel and unique transarterial (rather than transurethral) intervention. This is reflected in the differences between the UK (NICE), European (EAU), and American (AUA) guidelines, the latter of which only recommends the use of prostate artery embolization in a clinical trial setting. The main issues include questions over the duration of symptom response, cost-effectiveness, mechanism of action, patient selection, and other procedural technical considerations. These factors are the most pressing faced by proponents of prostate artery embolization, and we seek to highlight why their resolution is important to ensure men with benign prostate hyperplasia seeking a minimally invasive solution are optimally informed and most effectively managed.

**Main Points**

- Prostate artery embolization is a leading minimally invasive alternative to surgery.
- A transarterial, rather than transurethral, approach brings a unique set of indications and contraindications relative to other minimally invasive therapies for benign prostate hyperplasia.
- Current controversies include patient selection, duration of symptoms response, and its cost-effectiveness relative to surgery.
- Several procedural issues are also currently debated including management of pudendal collateral vessels, radiation dose minimization, and optimal equipment utilization.

## Introduction

Despite benign prostate hyperplasia (BPH) being one of the most common and debilitating diseases affecting men,^1^ viable minimally invasive alternatives to surgery have only emerged in the last few decades.^2^ These alternatives are driven by recent technological advances in multiple fields and together they promise to create a wealth of options for the 30 million men suffering from BPH worldwide.^3^ The aim is to widen the spectrum of patient choice and bridge the gap which previously existed between medication and surgery.^2^

One of the most promising of all minimally invasive therapies is prostate artery embolization (PAE). A wealth of evidence including randomized control trials^4,5^ and meta-analyses^6–8^ now support its use as a mainstream therapy for BPH, which is reflected by several national and international guidelines.^9–11^ Favorable to patients as a day case procedure, performed under local anesthetics, it preserves erectile function, fertility, does not require catheterization, involves minimal discomfort, has an excellent safety profile^12–14^ and it is one of the alternatives to prostatectomy in larger glands (>100 cm^3^).^15^ Furthermore, due to the entirely contrasting approach of PAE to other minimally invasive therapy/transurethral resection of the prostate (TURP) (transarterial vs transurethral), offering the option to patients is an important consideration for any urology service.

When compared with other minimally invasive surgical therapies (MISTs), a recent systematic review and meta-analysis^16^ identified PAE as a more effective treatment in terms of the International Prostate Symptom Score (IPSS) and the Quality of Life (QoL) scores over prostatic urethral lift (PUL) and water vapor (WV) thermal therapy. Only photoselective vaporization (PVP) scored higher in terms of these outcome parameters (random-effects model, IPSS at 12 months standard mean difference: PVP, −2.83; PUL, −1.62; WV −1.77; PAE, −2.61).

Despite the increasing popularity of PAE for patients with BPH, there remains several unresolved controversies which we aim to address in this review.

## Clinical and Research Consequences

### Mechanism of Action

Although devascularization of prostate is the clear instigator of symptom improvement in PAE, the exact mechanism by which devascularization causes symptom resolution remains unclear. Several concepts have since been proposed with varying levels of scientific support.^17,18^ Initially, several studies identified prostate volume reduction as correlating with symptom improvement,^19,20^ leading to the conjecture that volume reduction is the chief process of symptom resolution (demonstrated to be a mean of 40.4 ml volume reduction^13^). However further studies have found no significant association between prostate volume change and symptom improvement,^21,22^ and therefore this concept has subsequently largely been dismissed as overly simplistic.^23,24^ Also, against the idea of volume reduction being a causative process rather than an incidental association is the observation that prostate volume does not correlate well with symptom severity prior to any intervention.^25,26^ The concept that a volume reduction could therefore improve symptoms logically holds little water. Any volume reduction observed following PAE is likely to be an association seen in imaging, rather than the underlying mechanism for symptomatic relief.^18^

Perhaps the most compelling currently proposed mechanism of action is an impact on the “dynamic” component of BPH,^27^ the term given to escalated prostatic stiffness due to an increase in stromal smooth muscle tone. This increased tone is down due to the activation of alpha-receptors in the prostatic tissue urethra.^28^ The alpha-activation is triggered via sympathetic mediators in the local microcirculation.^28,29^ Promisingly several studies have confirmed an associated reduction in the elastic modulus of the prostate following PAE measured by several imaging techniques including magnetic resonance imaging (MRI).^30,31^ Alternatively, this reduction in stiffness could be sequelae of infarct remodeling with fibroblasts which reduce the prostatic stiffness,^32^ but either way, a reduction in the dynamic obstructive component is clearly a feasible action of PAE.

Understanding the detailed mechanism of action behind devascularization is not essential to PAE’s status as an effective treatment, as it meets established criteria for causation in all respects.^33^ However, further deciphering of the mechanism of action could enable optimization of the PAE technique and thus improve patient outcomes. More studies investigating the mechanism of symptom improvement are therefore welcome, in order to build on these theories put forward. For example, research into drug-loaded bead delivery which can optimize prostatic stiffness reduction (with alpha-receptor antagonist drug or an intermediate which promotes fibroblast remodeling) is an avenue that could benefit from additional research into the true method of PAE symptom improvement.

### Patient Selection

Several patient groups have been identified as a particularly promising target demographic for PAE.^34^ These include patients with large prostates (>80 ml), patients unfit for invasive treatment, those on anticoagulation, or sexually active patients looking for an alternative to medication.^34^ Considering the importance of shared decision-making with patients, the most important factor would be (and often is) a patient preference for PAE.^35^ Although PAE has been identified as a good option for these particular subgroups of men with BPH, which patients should be recommended PAE as a first-line intervention over TURP remains contentious and perhaps a subject of ongoing debate.

**Figure 1.**
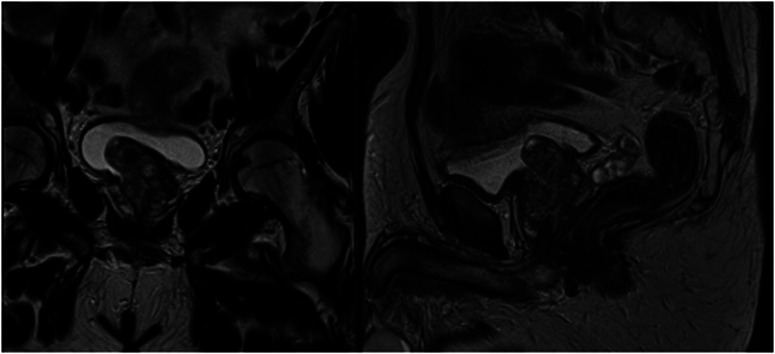
Intravesical protrusion of the prostate demonstrated on T2-weighted coronal and sagittal magnetic resonance image.

A further contentious point regarding patient selection for PAE is those patients with a large median lobe. This is an excluding feature for several other minimally invasive BPH therapies including UroLift and temporary implantable nitinol devices.^36^ The majority of studies have found that patients with a large median lobe protruding into the bladder (Figure 1) who undergo PAE have a good response to PAE,^37–40^ and the degree of median lobe protruding into the bladder can be successfully reduced,^40^ and severe intravesical prostatic protrusion has even been associated with improved symptom response after PAE.^39^ However, two studies have raised the possibility of a tall, mobile median lobe acting as a ball-valve mechanism^23,41^ flopping over to obstruct the bladder outflow. PAE would not be effective in this situation; however, neither would be another minimally invasive alternative.

A patient group well suited to PAE is those with hematuria in the presence of BPH and a contraindication to surgery. In this patient group, embolization is highly effective (up to 100%) if providing relief of hematuria.^42^

In terms of patient selection, when other minimally invasive treatments are taken into account, a clear treatment pathway becomes even more challenging. Further comparative studies on MISTs should therefore be considered to help patients identify which intervention strategy would suit them individually. A recent comparative study of minimally invasive treatments suggested PAE is the best treatment for the preservation of erectile function.^16^ If the sexual function is therefore particularly important to a patient, current evidence suggests PAE may therefore be the preferable invasive intervention.

### Optimal Equipment

PAE requires (among other equipment) two key pieces of specifically manufactured kit: a microcatheter and embolic particles. A variety of particle sizes and compositions are commercially available and sanctioned for use worldwide. Most embolic particles described for PAE incorporate polyvinyl alcohol (PVA), in either a calibrated spherical or a non-spherical (random “pop-corn” shape) form.^26,43^ Non-spherical PVA particles are known to “clump” together due to their irregular shape and therefore form a more proximal vascular occlusion.^44^ Both spherical and non-spherical particles have been described as safe and clinically effective for PAE.^45,46^ Several studies have also been conducted into the optimal particle for PAE, with a recent systematic review suggesting smaller particles yield a better outcome,^45^ which supports analysis of the UK-ROPE database that patients having an embolization with spherical particles of <300 µm have a greater symptom improvement.^47^ This is likely due to improved tissue infarction from more distal embolization preventing recruitment of alternative arterial pathways. One comparative study did identify a greater incidence of adverse events when smaller particles were used, but this was not statistically significant.^48^

### Radiation Dose

A disadvantage of PAE compared to transurethral intervention is the use of radiation during the procedure. The dose can be significant to both patient and operator, and therefore imperative that techniques to minimize radiation dose are always utilized. Although radiation dose during a standard PAE procedure does not reach the levels of deterministic harm (around 3 Gy),^49,50^ a consideration for any procedure involving radiation is the stochastic effects on patients (chance of malignancy related to the dose). The dose area product per PAE procedure is approximately 17 400 Gy/m^2^, which corresponds to an effective dose of approximately 47 mSv. In a patient population with an average age of 65, this is roughly equivalent to an additional lifetime cancer risk of 0.2% (baseline risk for men is 44.9%).^49^

**Figure 2.**
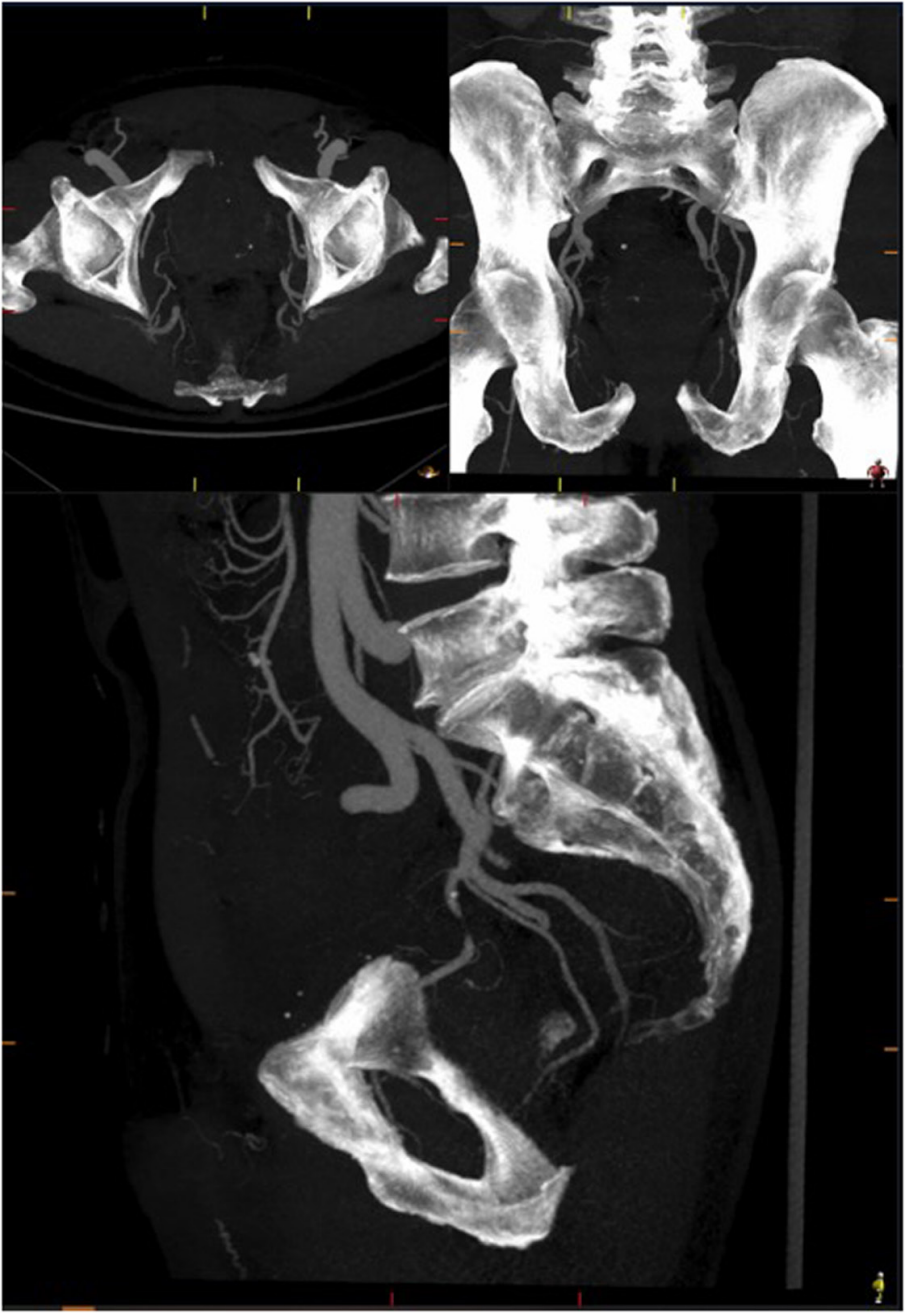
Planning computed tomography angiogram to identify the origin of the prostate arteries and facilitate procedural planning.

One controversial concept of dose reduction is the use of computed tomography (CT) angiography prior to the procedure.^51^ The technique exposes the patient to a further radiation dose, but it also gives a clear initial view of the anatomy (Figure 2), without which the procedural time and therefore dose could be prolonged. This is particularly pertinent in cases with an unusual origin of the prostatic artery, such as arising from a replaced obturator artery.^52,53^ Without a broad overview provided by prior CT angiography, the procedural dose when identifying the prostatic artery could be considerably higher.^51^ An alternative to CT is a planning MRI/MRA, which does not use radiation.^51,54–56^ However, although this modality is likely to identify significant anatomical variants, it provides a much lower spatial resolution and much more limited data regarding the anatomy^56^ (including difficulty in detecting features such as vessel anastomoses).

**Figure 3.**
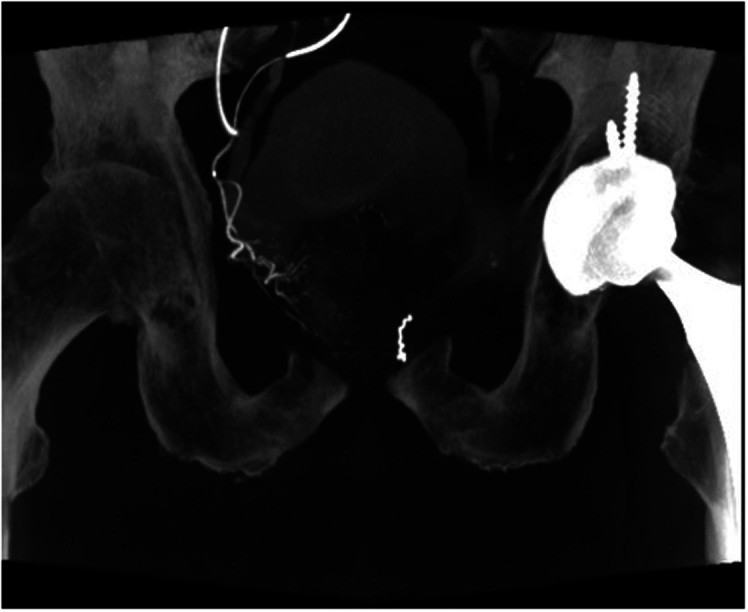
Three-dimensional cone beam computed tomography with contrast injection in the right prostate artery confirming gland enhancement (left side already treated with a protective coil placed at the base of the left lobe).

Cone beam CT is an important intraprocedural technique that has transformed several areas of interventional radiology (IR) practice.^57,58^ It allows a three-dimensional acquisition during the procedure with an associated dose penalty (Figure 3).^59^ How often and when to use it during a procedure is, therefore, a controversial issue to ensure a balance between radiation dose and that procedural safety/efficacy is maintained. If used sparingly and effectively, cone beam CT could also theoretically reduce the overall procedural dose to the patient as it provides valuable three-dimensional anatomical information,^43,60^ similar to CT angiography. However, if used ineffectually, cone beam CT can significantly increase the procedural dose to the patient (as it contributes almost 50% of the procedural dose to the patient).^46,61,62^

Finally, artificial intelligence software included with several leading IR suite manufacturers equipment includes an overlay of prior imaging, roadmap software, and automatic identification of the prostatic artery.^43,60,63,64^ These techniques hold promise in further reducing the dose of patients and practitioners by limiting the screening time required to cannulate the prostatic artery.^60^

### Duration of Symptom Response

The International Prostate Symptom Score is a validated and internationally accepted questionnaire to assess the symptoms of BPH.^65^ Along with the QoL score, it forms the basis of qualitative outcomes for BPH interventions, both invasive and non-invasive.^66^ Flow rate studies, such as maximum flow rate (*Q*_max_), from uroflowmetry can give an objective assessment of outcomes, but this is a proxy variable and not well correlated to symptomatic relief.^67^ A considerable number of prospective studies have demonstrated a significant symptom response (in both IPSS and QoL) following PAE including comparative and randomized studies^4,13,68,69^; however, a majority of these studies only follow up participants to 1 year. The current weakness within the evidence base for PAE is the limited number and quality of studies, which have been conducted for a greater period of follow-up.

Published patient series exist with up to 8 years of follow-up,^70–73^ all of which suggest a robust symptom response at a longer-term follow-up, which is undoubtedly promising. However, it should be noted these studies are conducted in tertiary world-renowned PAE centers, and the generalizability of these outcomes at low volume centers should be questioned. This is especially important as it is acknowledged that PAE is a technically challenging procedure with a steep and prolonged learning curve.^10,11,34^ For PAE to become acknowledged in all guidelines as a routine standard of care, better quality, and long-term prospective, comparative studies must be conducted. It should be noted that long-term evidence of other MISTs is also lacking, with no treatment close to rivaling the evidence base of TURP, which unanimously remains the gold standard of care.

### Treatment of Accessory Pudendal Arteries

**Figure 4.**
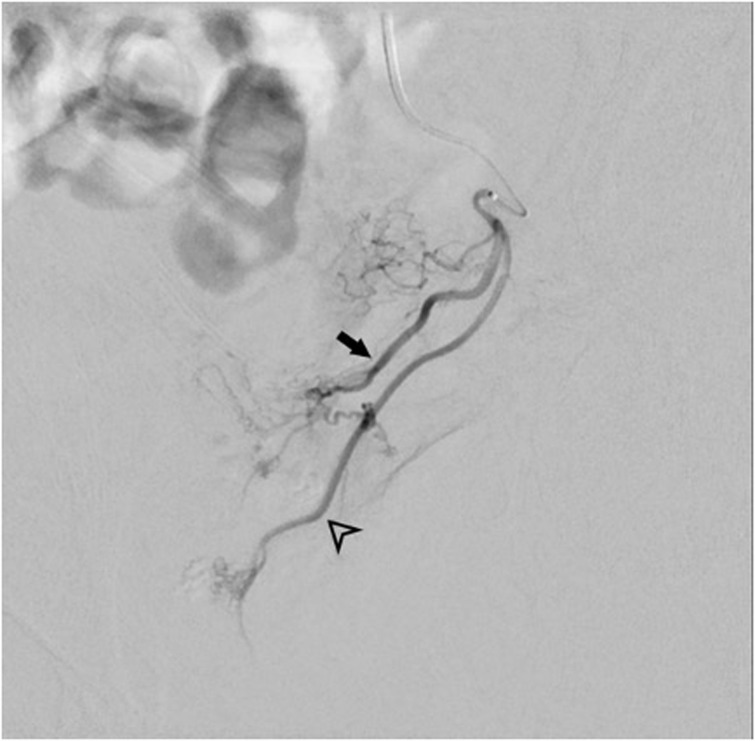
Contrast injection through a microcatheter in the left prostate artery demonstrates supply to the prostate (solid arrow) and accessory pudendal artery extending inferiorly below the prostate to the base of the penis (outlined arrowhead).

One of the strengths of PAE compared with transurethral surgery (including other minimally invasive therapies) is the sparing of sexual function.^16^ Many studies demonstrate that sexual function actually improves after PAE although this is probably due to the cessation of 5α-reductase inhibitors rather than a direct effect of embolization.^6–8^ The UK Register of Prostate Embolization (UK-ROPE) study demonstrated a mean improvement in The International Index of Erectile Function (IIEF) of 1.0 compared with a reduction in IIEF of 0.2 for TURP.^13^ Given this benefit of PAE, a controversy surrounds the management of anastomoses between the prostatic artery and penile vessels, usually via an accessory pudendal artery (Figure 4).^50^

Collateral vessels are a relatively common finding in PAE,^51^ which can be managed by protective coil embolization (preventing distal particle embolization), achieving a catheter position distal to the anastomotic vessel (or cannulating multiple small prostatic feeders), or utilizing flow redistribution via a balloon occlusion microcatheter.^12,42,74^ However, if the anastomotic vessel is an accessory pudendal artery to the penis, it may well have an important role in erection.^75–77^ Although coil embolization will protect distal particle embolization and end tissue ischemia,^78,79^ it could theoretically impair the ability to achieve an erection. No current consensus exists on whether accessory pudendal artery coiling is a safe practice.

### Cost-Effectiveness

As PAE is generally conducted as a day case without the need for general anesthetic or a recovery team, and with lower complication rates, it has a clear initial cost benefit over TURP per patient,^80^ especially in the first year.^81^ However, this superficial evaluation requires more detailed analysis as TURP holds established advantages in terms of better symptom response and subsequent QoL improvement.^6,13^ Furthermore, PAE cohorts have higher rates of re-intervention, including a considerable proportion (21% at 2 years^22^) going on to have TURP,^13,22^ which will impact on the cost-effectiveness of PAE. Furthermore, PAE cohorts have higher rates of re-intervention, including a considerable proportion (21% at 2 years^22^) going on to have TURP^13,22^ which will impact on the cost-effectiveness of PAE. A recent cost evaluation study, which took quality-adjusted life year into account, predicted a small overall greater cost for PAE compared with TURP.^28^ Further studies are therefore required to estimate the cost benefit/penalty to offering PAE to patients. As evidence also builds for other MIST BPH interventions,^29^ cost-effectiveness studies comparing these treatments among each other will also become increasingly important to enable healthcare providers to rationalize which treatments to offer.

## Conclusion

Prostate artery embolization (PAE) continues to emerge as a leading minimally invasive therapy for BPH, with a novel transarterial approach rather than the standard transurethral approach offered by all other invasive interventions for BPH. It therefore brings a unique set of advantages and disadvantages to the spectrum of treatment for BPH. The controversies addressed are the most pressing issues faced by the proponents of PAE and should be addressed as a priority. This is to ensure men with BPH are optimally informed and effectively managed as we navigate our way through a new era of minimally invasive treatments. Perhaps, centers should be able to offer multiple MIST options as a part of the treatment algorithm for BPH, with patient counseling and choice at the heart of these discussions.
